# Supplementary material for: Enter and Discuss Orders and Prescriptions (EPA 4): A Curriculum for Fourth-Year Medical Students
Source: MedEdPORTAL. 2022 Jul 5;18:11263. doi: 10.15766/mep_2374-8265.11263 (PMC9253226; doi:10.15766/mep_2374-8265.11263)
Supplement: Supplementary file 1 — Facilitator Guide.docxCase 1.docxCase 2.docxCase 1 Rubric.xlsxCase 2 Rubric.xlsxOrder Entry Workshop Debrief.pptxSelf-Report Confidence Instrument.docxGraduate Self-Report EPA 4 Preparedness Item.docx [file mep_2374-8265.11263-s001.zip › B. Case 1.docx]

**Workshop Case #1 Template**

This document includes the necessary information to create an encounter in the electronic heath record educational environment.

**Setting:**

Emergency Department

**Patient Profile:**

Name: Robyn Granger

Age: 18

Gender: female

**Allergies:**

Penicillin - Reaction anaphylaxis

**Code Status:**

Full Code

**Reason for Visit**:

Fever and vomiting

**Vital Signs:**

Height: 5’4”

Weight 135 lbs

**Time ED Arrival Admission** *(2 hours later)*

Temperature 101.3 F 99.3 F

Blood Pressure 117/68 mmHg 115/65 mmHg

Heart Rate 110 89

Respiratory Rate 18 14

Oxygen Saturation 99% on room air 99% on room air

**History:**

Past Medical History: Anaphylaxis when treated with amoxicillin for otitis media as a young child

Past Surgical History: None

Family History: Mother – Hypertension

Father – Nephrolithiasis

Social History: Tobacco Products – Never used

Alcohol – occasional beer with friends. 1-2 drinks at a time.

Recreational Drugs – Never used

Sexual History – 1 current male partner (boyfriend)

**Home Medications:**

| Norgestimate-ethinyl estradiol | 0.25 mg-35mcg | oral | once daily |
| --- | --- | --- | --- |
| Ibuprofen | 400mg | oral | every 6 hours as needed |

**Labs** *(admit minus 1 hour):*

Urinalysis: Specific Gravity 1.030 (high); Leukocyte esterase (positive); Nitrites (positive); Heme (positive); Protein (positive, 1+); glucose (negative); White Blood Cells 65, Red Blood Cells 50

CBC: WBC 12.5 Hb 12 Plt 335 Diff Pending

Chemistry: Na 142 K 3.8 Cl 110 HCO3 22 BUN 22 Cr 1.0 Gluc 87

Urine culture: pending

Blood culture: pending

Urine HCG: Negative

**Imaging** *(admit minus 1 hour):*

CT abdomen and pelvis

Name: Robyn Granger

Exam Date: Current date

Comparison: None

Indication: Right flank pain

Technique: CT abdomen and Pelvis without Intravenous Contrast

Findings:

The liver is unremarkable. Gallbladder visualized. There is no ductal dilatation. Pancreas unremarkable without ductal dilatation. Spleen normal in size. Adrenals unremarkable without mass. Left kidney and ureters unremarkable. 7mm obstructing stone in the right ureter with associated hydronephrosis of the right kidney. There is some associated perinephric stranding visualized on the right. No visible abscess. The bladder is unremarkable without stones. Reproductive organs unremarkable. Stomach and bowel unremarkable without evidence of obstruction or mucosal thickening. A normal appearing appendix is visualized.

Impression: 7 mm obstructing stone with associated hydronephrosis of the right kidney.

**Emergency Department Orders:**

Place / Maintain Peripheral IV

0.9% sodium chloride 1,000mL bolus

Ondansetron 4mg IV once

Morphine 2mg IV once

CT abdomen and pelvis without IV contrast

Urinalysis with microscopy

Urine culture

CBC with differential

Chem 7

Peripheral blood culture x 2

Urine HCG

**Emergency Department Documentation:**

**Nurse Triage Note** *(admission time minus 2 hours)*: Patient reports she was diagnosed with kidney stone 3 days ago. Now with fever and vomiting.

**Nurse Note** *(admission time minus 1.5 hours):* PIV placed. ED resident to the bedside. Normal saline bolus started. Patient instructed on urine sample collection

**Nurse Note** *(admission time minus 1.25 hours):* Pt with large emesis. Order for antiemetic and pain control requested.

**Nurse Note** *(admission time minus 1 hour):* Ondansetron 4mg IV and Morphine 2 mg IV administered. Labs obtained. Patient to CT scan.

**ED Physician Note** *(admission time):*

Chief Complaint: Fever and Vomiting

History of Present Illness: 18-year-old female with no significant medical history presents with fever and vomiting. Patient reports she was well until about 3 days ago when she developed intermittent right flank pain. She presented to her PCP at that time where a urine sample was positive for blood. She was diagnosed with suspected nephrolithiasis, instructed to strain her urine, use OTC pain control meds, drink fluids, and follow-up. Yesterday she developed dysuria, worsening/more constant right flank pain, and fevers up to 101.4. She also developed nausea with vomiting, and an inability to tolerate oral intake. When she contacted her PCP, she was instructed to come to the emergency department for further evaluation.

Past Medical History: None

Home Medications:

Norgestimate-ethinyl estradiol tablet 0.25mg-35mcg (orthocyclen) 1 tab po daily

Ibuprofen 400mg Q6H PRN Pain

Allergies: Amoxicillin/Penicillins - anaphylaxis

Social History: Occasional ETOH intake. No recreational drug use or tobacco use

Family History: Mother with hypertension. Father with hx of kidney stones

Review of Systems: Positive for fever, nausea, vomiting, right flank pain, dysuria. Decreased appetite. Otherwise negative.

Physical Exam:

Initial Vitals: T 101.3 P 110 BP 117/68 RR 18 O2Sat 99% on RA Wt 135 lb Ht 5ft 4 in BMI 23.7

Most Recent Vitals: T 99.3 P 89 BP 115/65 RR 14 O2 Sat 99% on RA

General: Awake, alert, age-appropriate female. Visibly uncomfortable, though answers questions appropriately

HEENT: No conjunctival injection. No mucosal lesions. Mucous membranes dry.

CV: Tachycardic with regular rhythm. No murmurs. No LE Edema.

Lungs: CTA B. Easy work of breathing. No focal abnormalities.

Abdomen: Soft, suprapubic tenderness to palpation though no peritoneal signs. Normal bowel sounds. No organomegaly.

GU: Right flank with marked tenderness to palpation. Left flank non-tender.

Neuro: Ambulates. No focal abnormalities. Oriented x 3

Labs:

Urinalysis: Specific Gravity 1.030 (high); Leukocyte esterase (positive); Nitrites (positive); Heme (positive); Protein (positive, 1+); glucose (negative); White Blood Cells 65, Red Blood Cells 50

CBC: WBC 12.5 Hb 12 Plt 335 Diff Pending

Chemistry: Na 142 K 3.8 Cl 110 HCO3 22 BUN 22 Cr 1.0 Gluc 87

Urine culture: pending

Blood culture: pending

Urine HCG: Negative

Imaging:

CT abdomen and Pelvis without Intravenous Contrast

Findings:

The liver is unremarkable. Gallbladder visualized. There is no ductal dilatation. Pancreas unremarkable without ductal dilatation. Spleen normal in size. Adrenals unremarkable without mass. Left kidney and ureters unremarkable. 7mm obstructing stone in the right ureter with associated hydronephrosis of the right kidney. There is some associated perinephric stranding visualized on the right. No visible abscess. The bladder is unremarkable without stones. Reproductive organs unremarkable. Stomach and bowel unremarkable without evidence of obstruction or mucosal thickening. A normal appearing appendix is visualized.

Impression: - 7 mm obstructing stone with associated hydronephrosis of the right kidney.

Assessment and Plan:

18-year-old female presents with right sided nephrolithiasis with associated hydronephrosis, complicated by acute pyelonephritis

1. Nephrolithiasis with associated urinary obstruction – May require urologic intervention in context of acute infection. Admitting medical team to determine plan of care with consideration for urology consult. Pain control.
2. Acute pyelonephritis – Admitting team to start empiric antibiotic therapy and follow-up culture data to guide adjustment of antibiotic therapy. Urine and blood cultures obtained, pending.
3. Nausea and vomiting – IV antiemetics PRN
4. Dehydration – IV fluid hydration.
5. Full code. Personally verified with patient.

**Order Set Considerations:**

Students should have access to institution-specific order sets relevant to general medical/surgical patients, as well as order sets specific to acute pyelonephritis and/or nephrolithiasis if they exist.

**Additional Resources:**

Students should have access to institution-specific evidence-based practice guidelines, including those for acute pyelonephritis, nephrolithiasis, and deep venous thrombosis prophylaxis.
