# Supplementary material for: Enter and Discuss Orders and Prescriptions (EPA 4): A Curriculum for Fourth-Year Medical Students
Source: MedEdPORTAL. 2022 Jul 5;18:11263. doi: 10.15766/mep_2374-8265.11263 (PMC9253226; doi:10.15766/mep_2374-8265.11263)
Supplement: Supplementary file 1 — Facilitator Guide.docxCase 1.docxCase 2.docxCase 1 Rubric.xlsxCase 2 Rubric.xlsxOrder Entry Workshop Debrief.pptxSelf-Report Confidence Instrument.docxGraduate Self-Report EPA 4 Preparedness Item.docx [file mep_2374-8265.11263-s001.zip › C. Case 2.docx]

**Workshop Case #2 Template**

This document includes the necessary information to create an encounter in the electronic heath record educational environment

**Setting:**

Emergency Department

**Patient Profile:**

Name: Brian Smith

Age: 33

Gender: male

**Allergies:**

No known drug allergies

**Code Status:**

Full Code

**Reason for Visit**:

Fainting and diarrhea

**Vital Signs:**

Height: 5’7”

Weight 180 lb

**Time ED Arrival Admission** *(3 hours later)*

Temperature 99.6 F 98.9 F

Blood Pressure 102/69 mmHg 110/70 mmHg

Heart Rate 118 89

Respiratory Rate 22 20

Oxygen Saturation 98% on room air 100% on room air

**History:**

Past Medical History: Crohn’s disease diagnosed at age 31

GERD

C. diff colitis – 5 months ago

Past Surgical History: None

Family History: Mother – Hypertension

Father – hypercholesterolemia

Type II diabetes mellitus

Social History: Tobacco Products – Never used

Alcohol – 4 drinks / week

Recreational Drugs – Never used

Sexual History – 1 current female partner (wife).

**Home Medications:**

| 6-Mercaptopurine | 100mg | oral | once daily |
| --- | --- | --- | --- |
| Omeprazole | 20mg | oral | once daily |

**Labs** *(admit minus 1 hour and 15 minutes):*

WBC 18.3      Hgb 10.8    Plts 312 Diff Pending

Na 149     K 3.8     Cl 111      Bicarb 19     BUN 20     Cr 1.4 Gluc 72 Mg 1.8

D-Dimer: 0.9

Type and cross obtained

**Imaging** *(admit minus 1 hour):*

Abdominal Plain Film

Name: Brian Smith

Exam Date: Current date

Comparison: None

Indication: Diarrhea, Crohn’s disease

Technique: Single view of the abdomen

Findings:

Lung bases are clear. Boney structures appear normal. No evidence of free air within the abdomen. There is a normal bowel gas pattern without evidence of ileus or obstruction. No abnormal calcifications or evidence of mass effect.

Impression: Normal exam

**Emergency Department Orders:**

Place / Maintain Peripheral IV

0.9% sodium chloride 1,000mL bolus

CBC with differential

Chem 10

D-dimer

Type and cross

Abdominal x-ray

EKG

**Emergency Department Documentation:**

**Nurse Triage Note** *(admission minus 3 hours):*  Patient brought to the ED by girlfriend after fainting episode.  Currently alert and oriented.

**Nurse Note** *(Admission minus 2 hours and 15 min):* Patient with loose blood-streaked stool.  ED resident to the bedside.

**Nurse Note** *(Admission minus 1 hour and 15 min):*  Patient with second loose blood-streaked stool.  IV placed.  Labs sent.  Normal saline bolus started.

**Nurse Note** *(admission minus 30 min):*  Patient unsteady on ambulating to restroom. Assistance provided.

**ED Physician Note** *(admission time):*

Chief Complaint: fainting and diarrhea

History of Present Illness:  33 year old male with history of Crohn’s disease on immunosuppression presented following fainting episode at grocery store.  Patient was waiting in line for check out, became dizzy/light-headed, and had brief loss of consciousness.  Witnessed fall, caught by girlfriend. Did not hit head.

At baseline has an estimated 4 loose stools daily. In past week notes increased frequency of blood-streaked loose stools, up to 10 times daily. No vomiting. Endorses crampy abdominal discomfort which is relieved with bowel movements. Poor appetite, and has been having difficulty maintaining PO.  No known sick contacts or exposures. No recent courses of antibiotics.

Past Medical History:

Crohn’s disease diagnosed at 31 years of age.  No bowel resections.

History of clostridium difficile 5 months ago treated with oral vancomycin

GERD

Allergies:  None

Home Medications:  6-mercaptopurine 100mg po daily; omeprazole 20 mg po daily

Social Hx:

Sexually active, one female partner.  4 drinks/wk. No tobacco. No illicit substances

Family hx:

Mom with HTN, father with hypercholesterolemia and DM, younger brother healthy

No family history of sudden cardiac death

Review of systems:

As above. No fevers or chills. Denies dyspnea. No recent weight loss.

Vitals:

Initial Vitals: T 99.6 P 118 BP 102/69 RR 22  O2Sat 98% on RA. Ht 5/7’’ Wt 180lb

Most Recent Vitals: T 98.9 P 89 BP 110/70 RR 20 O2 sat 100% on RA

Physical Exam:

General: Alert, interactive. Observed walking to the bathroom with some unsteadiness.

Mouth: dry lips, MM tachy

Lungs: CTA B

CV: Tachycardic, regular.  No murmurs. No JVD.

Abd: hyperactive bowel sounds, soft, mildly tender to palpation diffusely, no guarding or rebound tenderness. Liver not palpated.

Ext: Strong pulses, no edema

Neuro: non-focal

Labs :

WBC 18.3      Hgb 10.8    Plts 312 Diff Pending

Na 149     K 3.8     Cl 111      Bicarb 19     BUN 20     Cr 1.4 Gluc 72 Mg 1.8

D-Dimer: negative

Type and cross obtained

Imaging:

Abdominal x-ray- no free air, non-obstructive bowel gas pattern.

EKG: Personally reviewed. Sinus tachycardia.  Otherwise normal.

Assessment and Plan:

33 y/o M with hypovolemic syncope, orthostatic symptoms secondary to increased stool loses.  Also with increased frequency of bloody stools. Ddx includes acute flare of inflammatory bowel disease vs clostridium difficile colitis, vs other enteritis (viral vs bacterial vs other).

- 2 large bore PIVs placed and NS bolus given
- Type and cross obtained
- admit to gen med for hydration and further evaluation of etiology of bloody diarrhea
- Note, patient is followed closely with OSU Gastroenterology re his IBD and requests that they be involved in his care during inpatient stay.
- Personally verified pt's preferred code status of Full Code

**Order Set Considerations:**

Students should have access to institution-specific order sets relevant to general med/surg patients, as well as any order sets specific to inflammatory bowel disease, syncope, or diarrhea.

**Additional Resources:**

Students should have access to institution-specific evidence based practice guidelines, including those for syncope, inflammatory bowel disease, clostridium difficile, and deep venous thrombosis prophylaxis.
