# Supplementary material for: Enter and Discuss Orders and Prescriptions (EPA 4): A Curriculum for Fourth-Year Medical Students
Source: MedEdPORTAL. 2022 Jul 5;18:11263. doi: 10.15766/mep_2374-8265.11263 (PMC9253226; doi:10.15766/mep_2374-8265.11263)
Supplement: Supplementary file 1 — Facilitator Guide.docxCase 1.docxCase 2.docxCase 1 Rubric.xlsxCase 2 Rubric.xlsxOrder Entry Workshop Debrief.pptxSelf-Report Confidence Instrument.docxGraduate Self-Report EPA 4 Preparedness Item.docx [file mep_2374-8265.11263-s001.zip › F. Order Entry Workshop Debrief.pptx]

## Slide 1
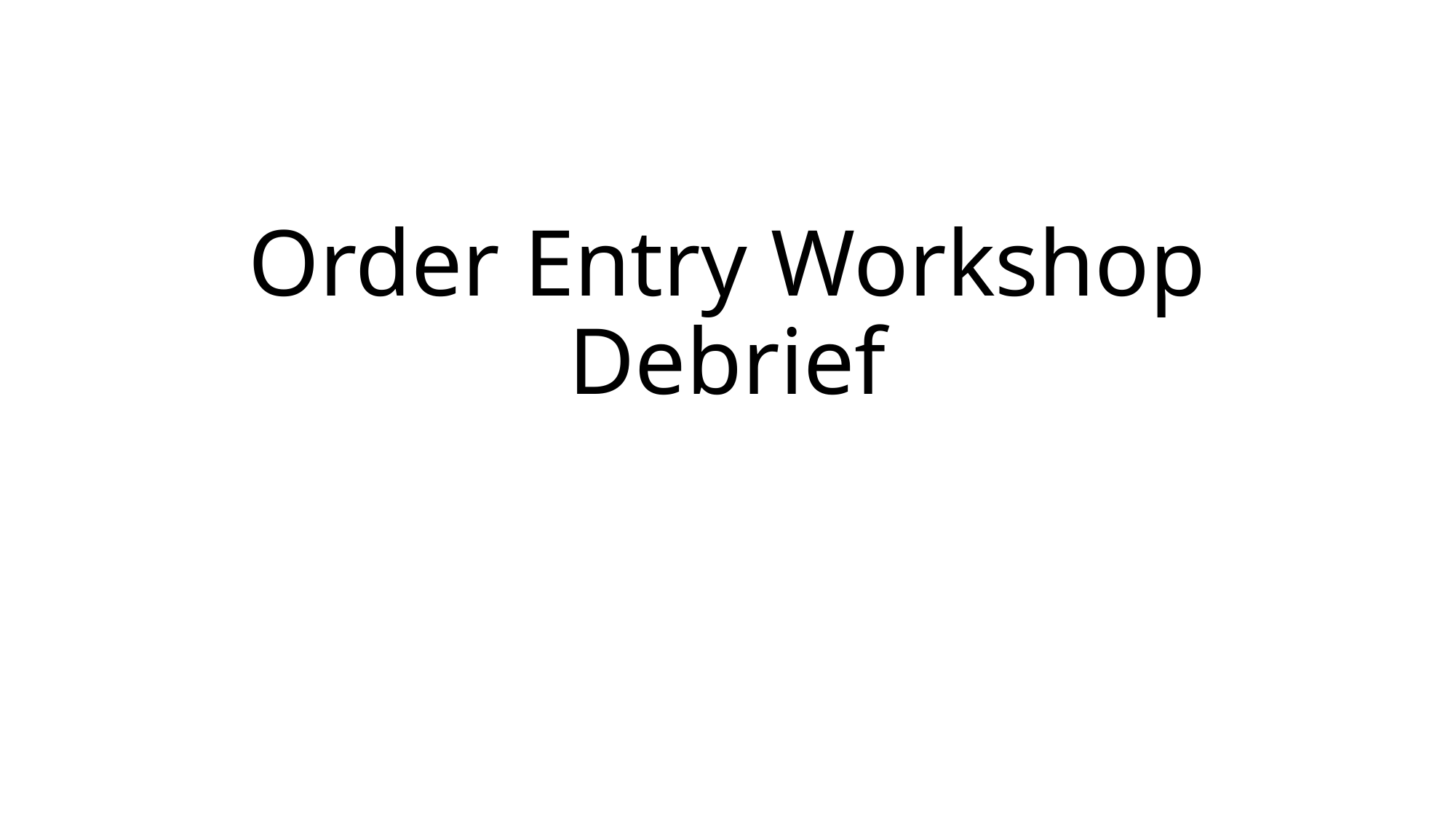

# Order Entry Workshop Debrief

## Slide 2
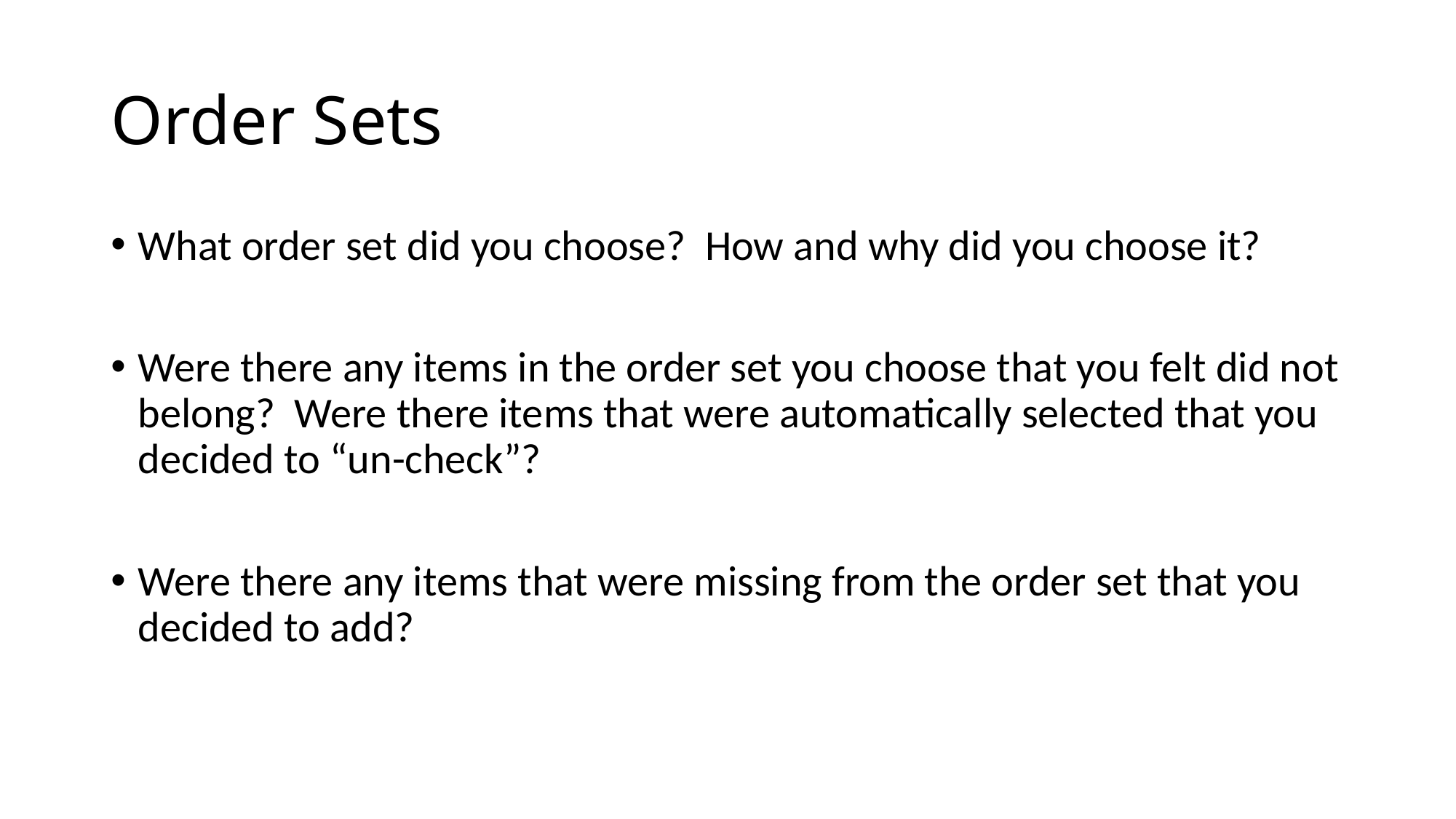

# Order Sets
What order set did you choose? How and why did you choose it?
Were there any items in the order set you choose that you felt did not belong? Were there items that were automatically selected that you decided to “un-check”?
Were there any items that were missing from the order set that you decided to add?

## Slide 3
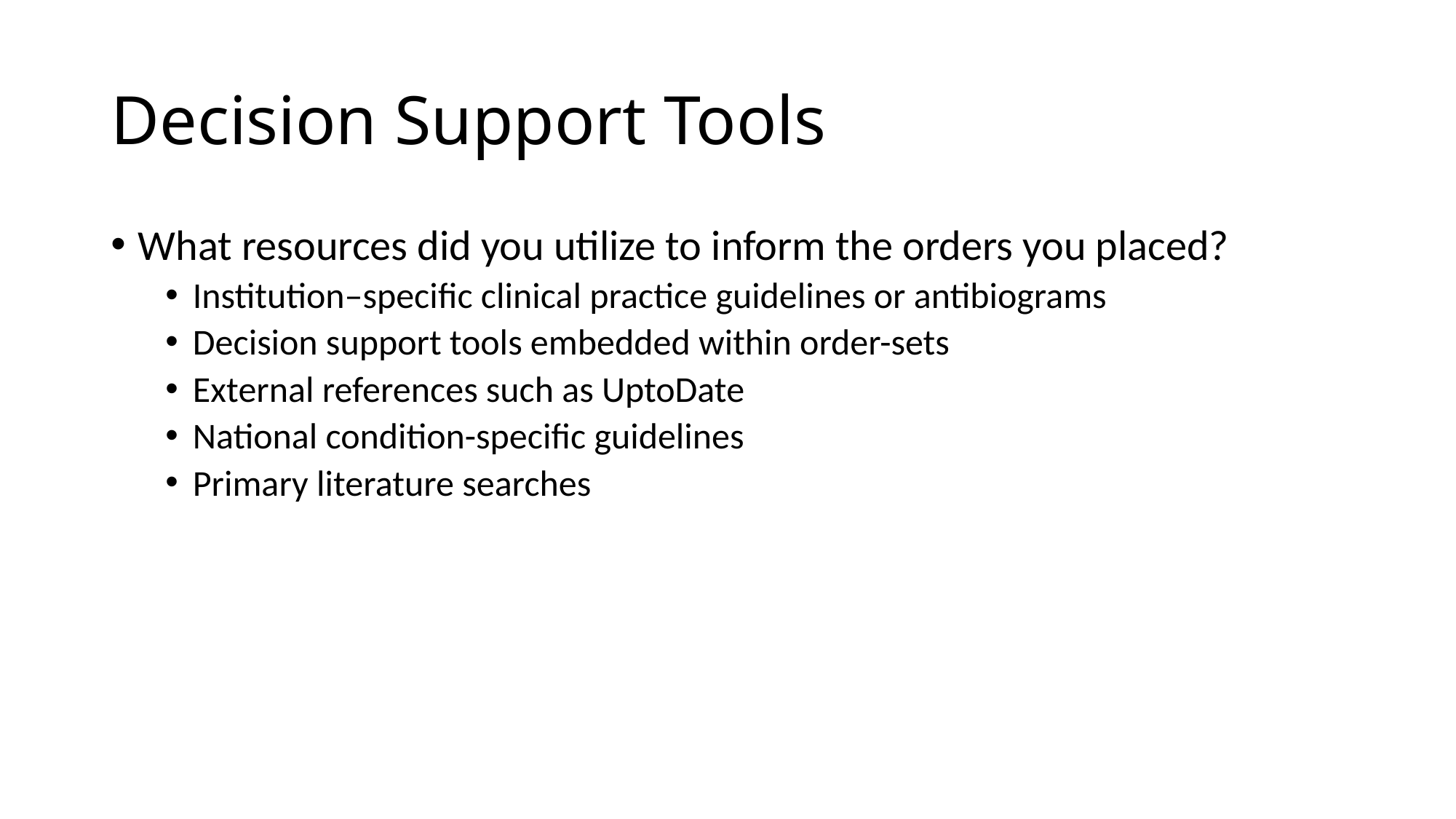

# Decision Support Tools
What resources did you utilize to inform the orders you placed?
Institution–specific clinical practice guidelines or antibiograms
Decision support tools embedded within order-sets
External references such as UptoDate
National condition-specific guidelines
Primary literature searches

## Slide 4
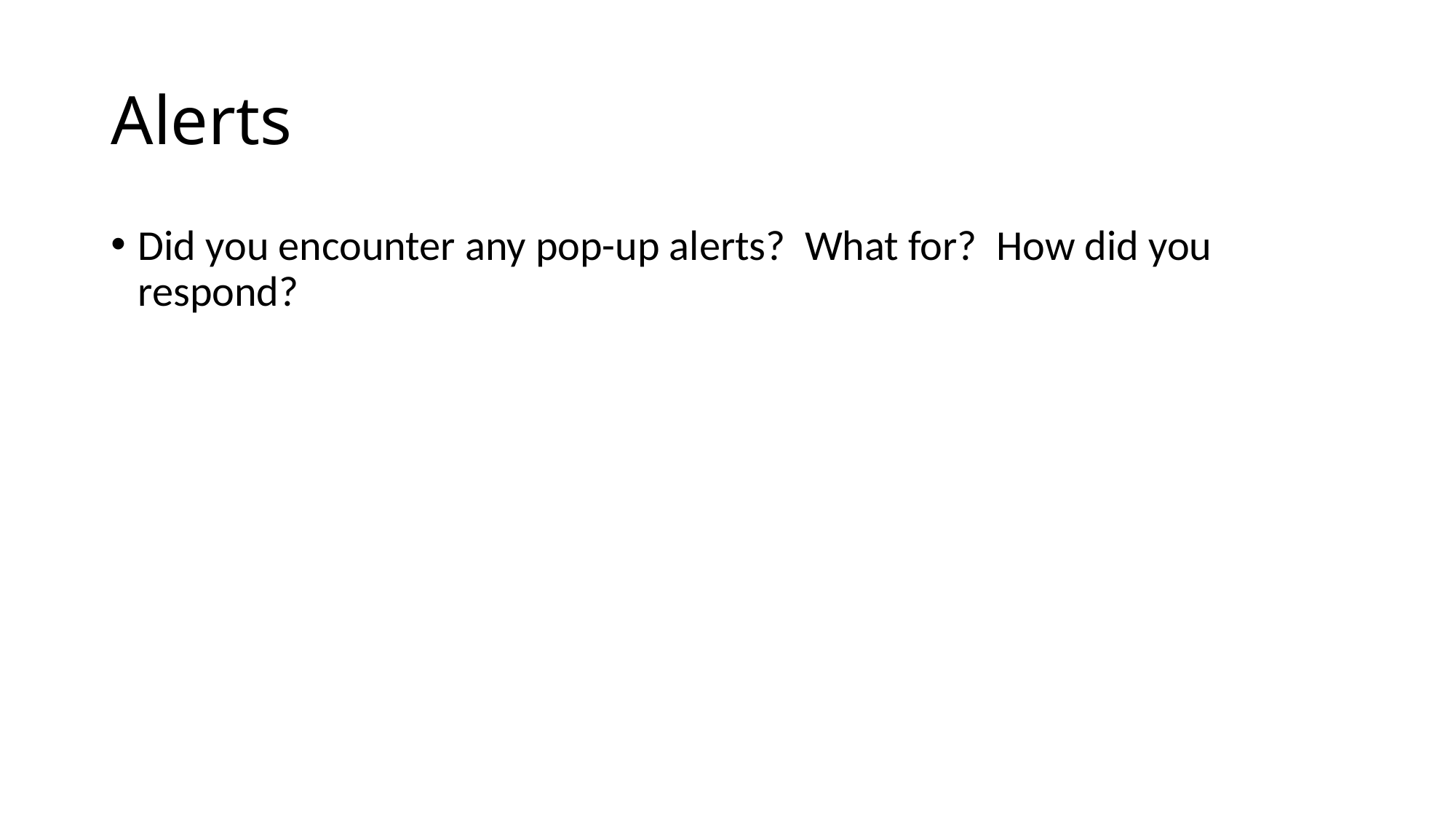

# Alerts
Did you encounter any pop-up alerts? What for? How did you respond?

## Slide 5
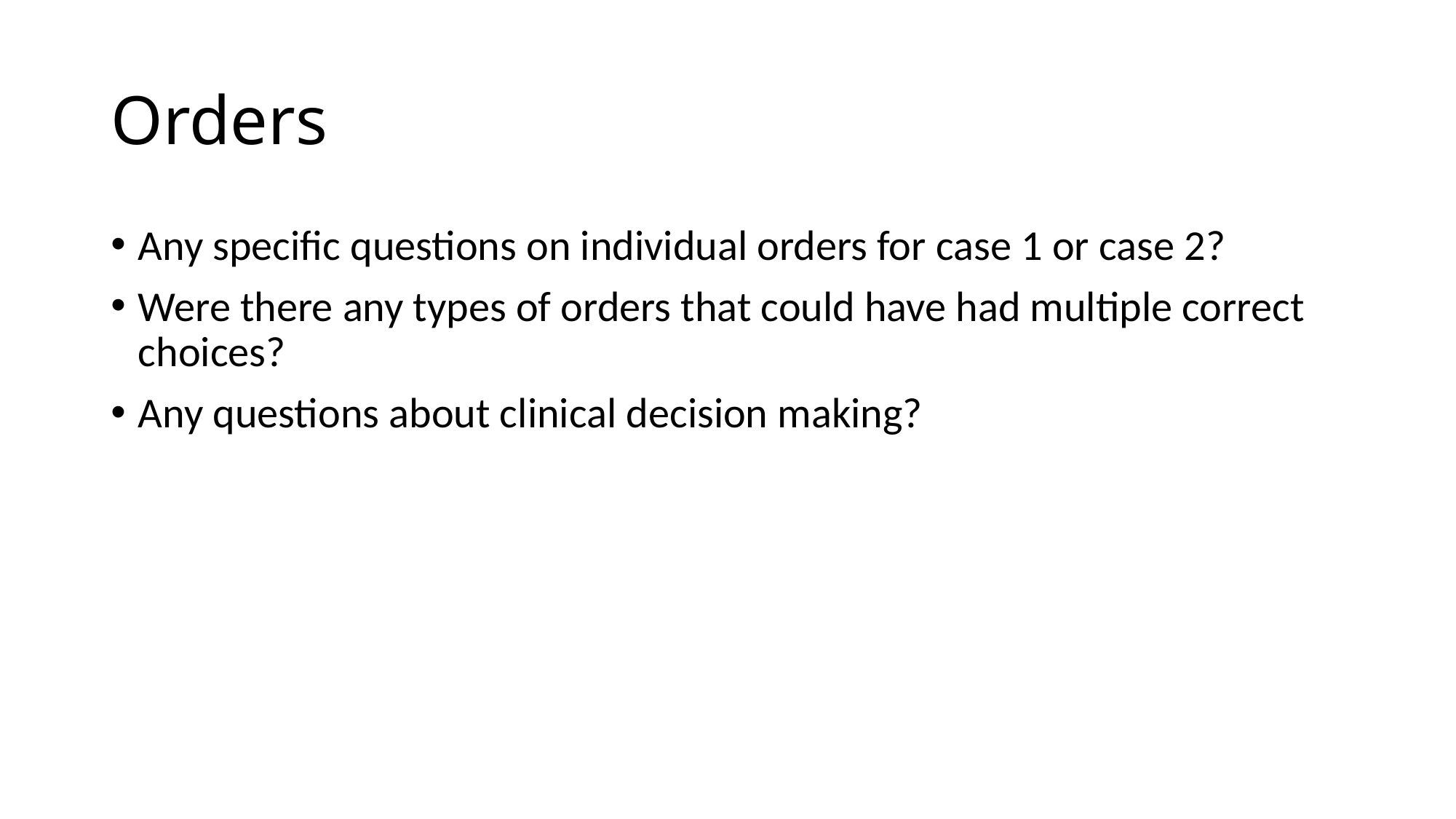

# Orders
Any specific questions on individual orders for case 1 or case 2?
Were there any types of orders that could have had multiple correct choices?
Any questions about clinical decision making?

## Slide 6
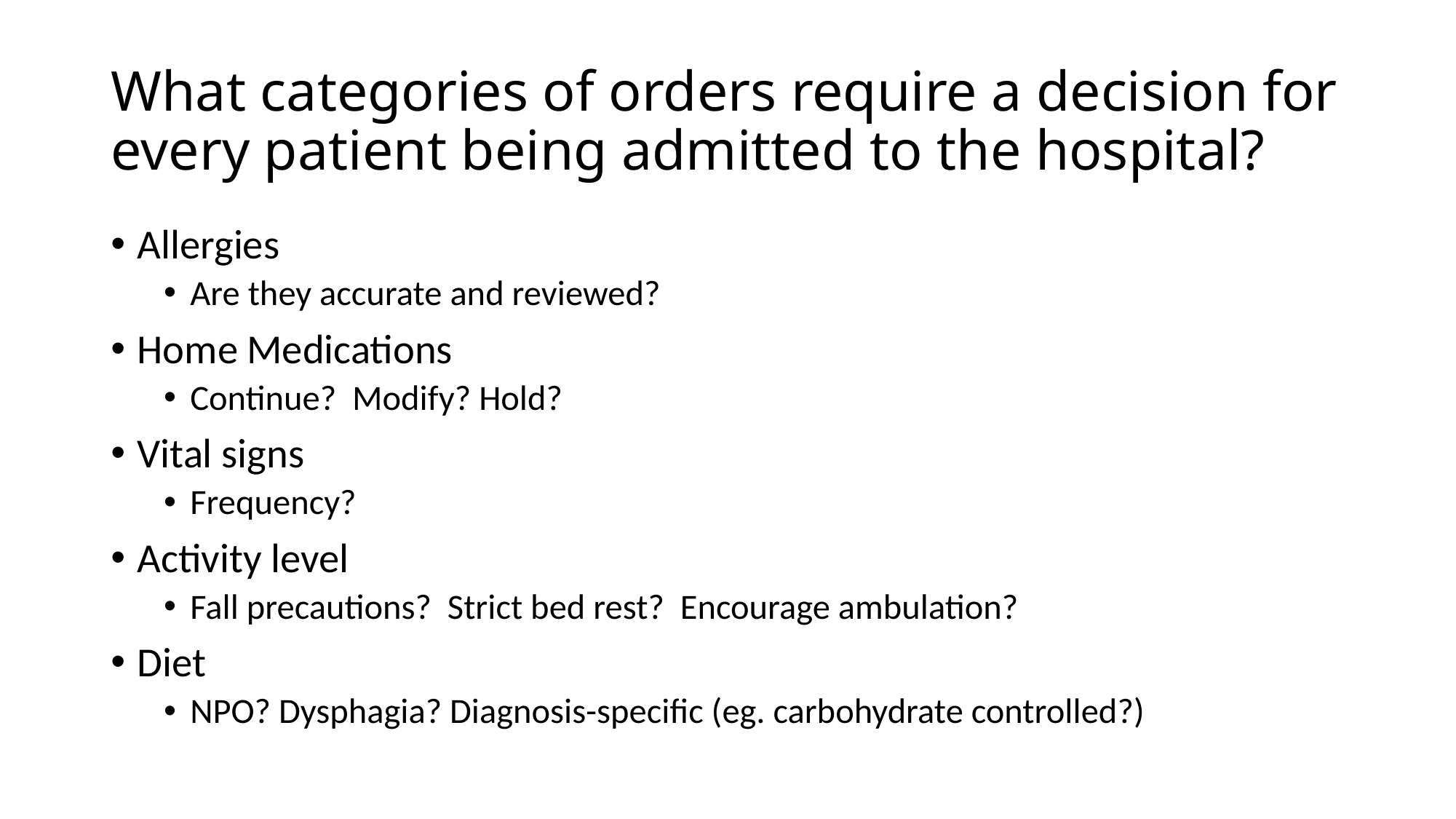

# What categories of orders require a decision for every patient being admitted to the hospital?
Allergies
Are they accurate and reviewed?
Home Medications
Continue? Modify? Hold?
Vital signs
Frequency?
Activity level
Fall precautions? Strict bed rest? Encourage ambulation?
Diet
NPO? Dysphagia? Diagnosis-specific (eg. carbohydrate controlled?)

## Slide 7
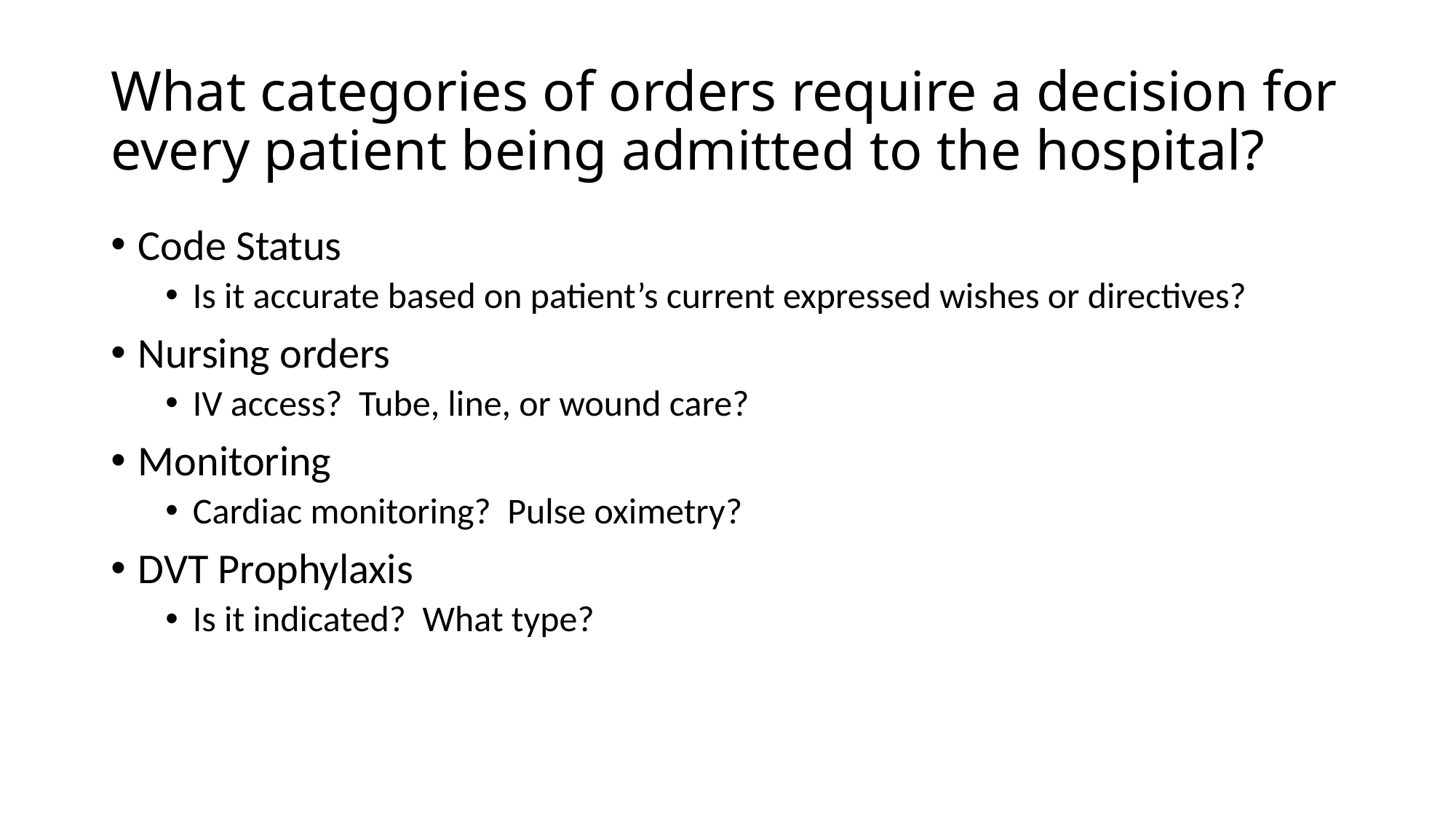

# What categories of orders require a decision for every patient being admitted to the hospital?
Code Status
Is it accurate based on patient’s current expressed wishes or directives?
Nursing orders
IV access? Tube, line, or wound care?
Monitoring
Cardiac monitoring? Pulse oximetry?
DVT Prophylaxis
Is it indicated? What type?

## Slide 8
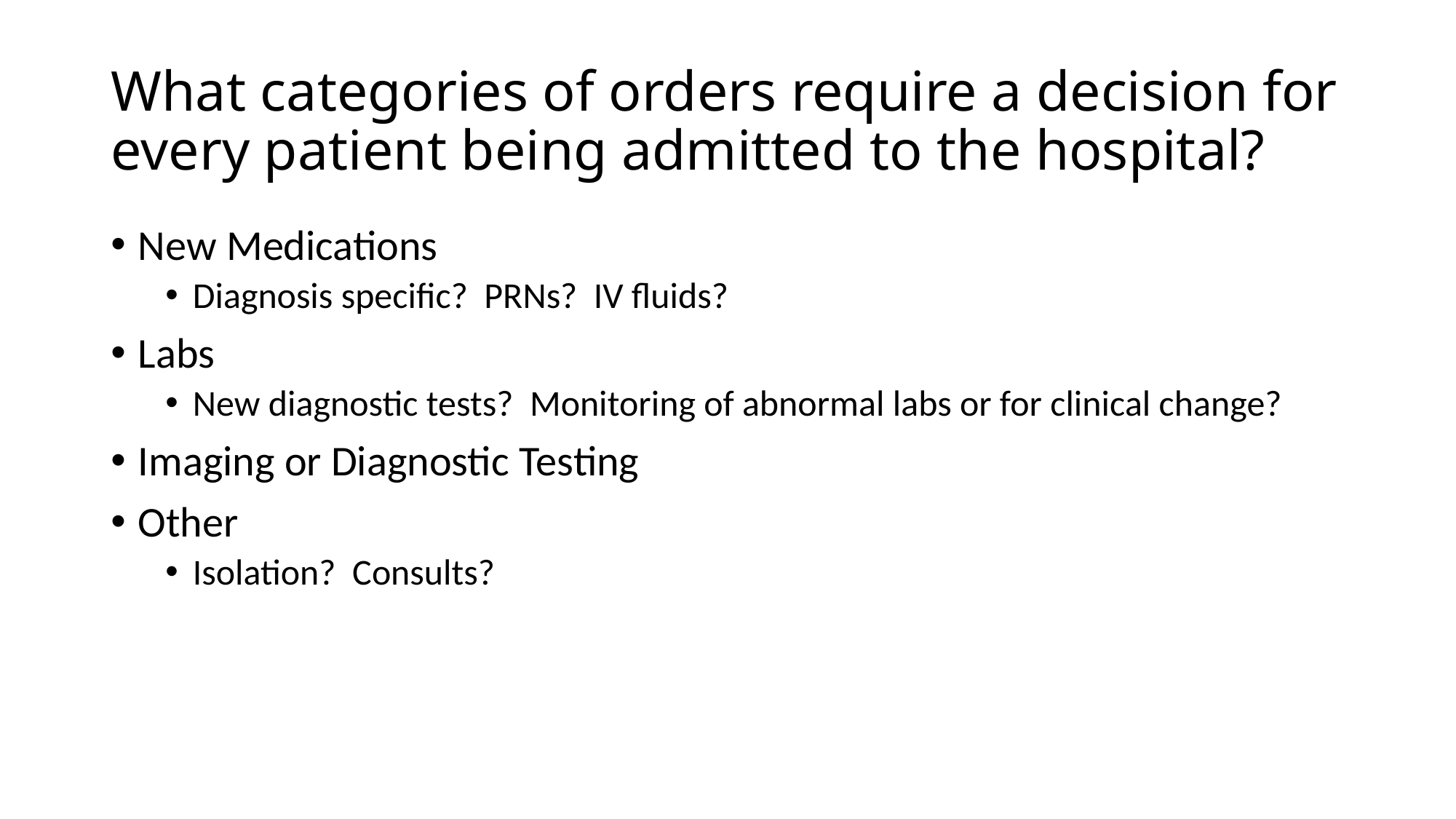

# What categories of orders require a decision for every patient being admitted to the hospital?
New Medications
Diagnosis specific? PRNs? IV fluids?
Labs
New diagnostic tests? Monitoring of abnormal labs or for clinical change?
Imaging or Diagnostic Testing
Other
Isolation? Consults?

## Slide 9
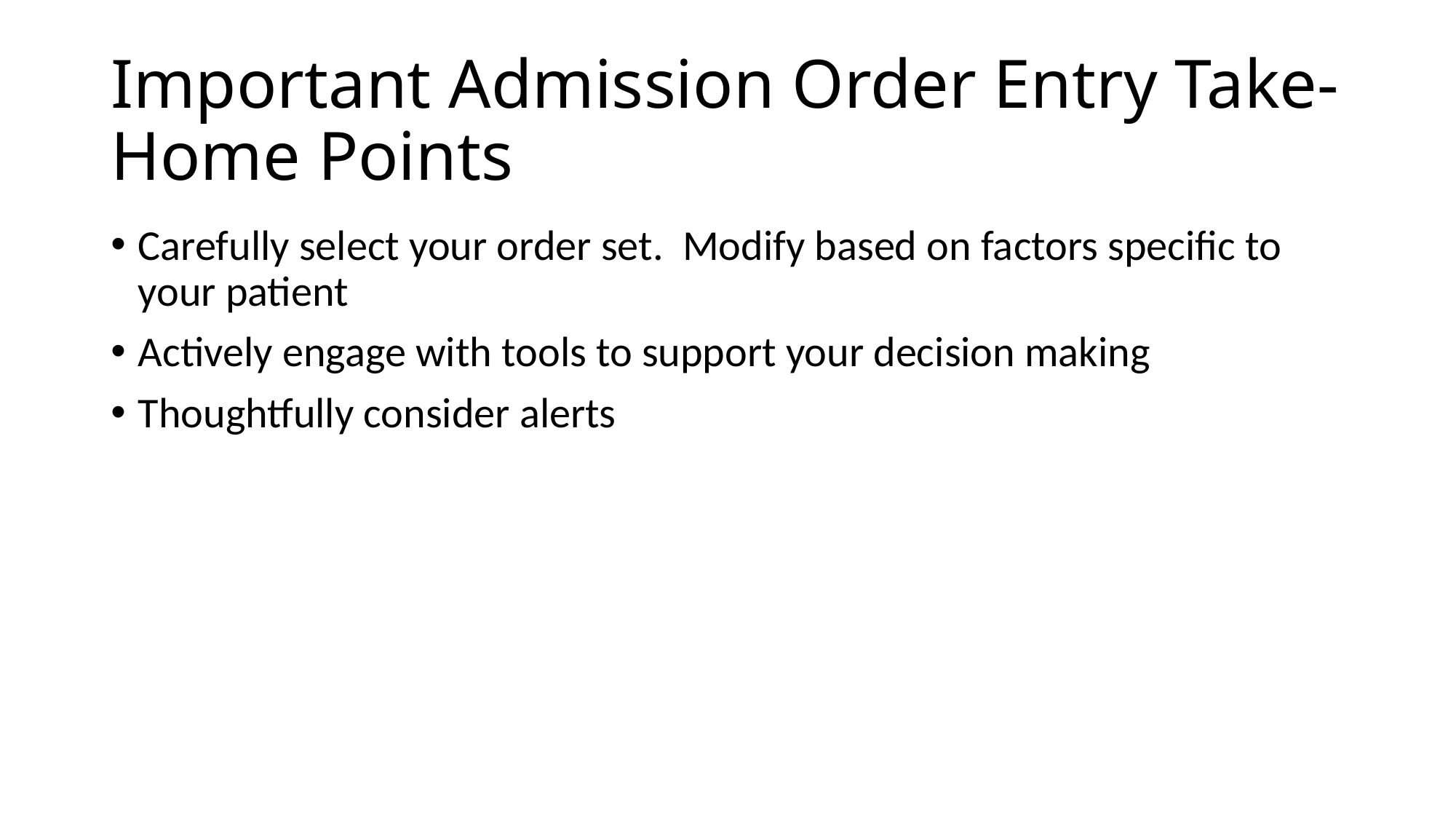

# Important Admission Order Entry Take-Home Points
Carefully select your order set. Modify based on factors specific to your patient
Actively engage with tools to support your decision making
Thoughtfully consider alerts
