# Supplementary material for: Enter and Discuss Orders and Prescriptions (EPA 4): A Curriculum for Fourth-Year Medical Students
Source: MedEdPORTAL. 2022 Jul 5;18:11263. doi: 10.15766/mep_2374-8265.11263 (PMC9253226; doi:10.15766/mep_2374-8265.11263)
Supplement: Supplementary file 1 — Facilitator Guide.docxCase 1.docxCase 2.docxCase 1 Rubric.xlsxCase 2 Rubric.xlsxOrder Entry Workshop Debrief.pptxSelf-Report Confidence Instrument.docxGraduate Self-Report EPA 4 Preparedness Item.docx [file mep_2374-8265.11263-s001.zip › G. Self-report Confidence Instrument.docx]

**Self-report Confidence Instrument (An instrument derived from the modified Chen entrustment scale^19^)**

Pre-workshop:

1. Which statement best reflects your current confidence level with entering admission orders in the electronic medical record (EMR)?
   1. I have watched others place admission orders but do not feel comfortable doing so by myself.
   2. I would feel comfortable placing admission orders synchronously with my resident or attending (We place the orders together).
   3. I would feel comfortable placing admission orders by myself with constant supervision (The resident or attending watches me place all the orders).
   4. I would feel comfortable placing admission orders independently (to be pended and co-signed by the resident or attending later).
   5. If I had the EMR clearance to place orders independently, I would feel comfortable entering admission orders without someone double checking (no co-sign necessary).

Post-workshop:

1. Which statement best reflects your confidence level with entering admission orders in the electronic medical record (EMR) after the admission order entry workshop?
   1. I have watched others place admission orders but do not feel comfortable doing so by myself.
   2. I would feel comfortable placing admission orders synchronously with my resident or attending (We place the orders together).
   3. I would feel comfortable placing admission orders by myself with constant supervision (The resident or attending watches me place all the orders).
   4. I would feel comfortable placing admission orders independently (to be pended and co-signed by the resident or attending later).
   5. If I had the EMR clearance to place orders independently, I would feel comfortable entering admission orders without someone double checking (no co-sign necessary).

Post-clinical course:

1. When admitting a new patient, which statement best reflects your confidence level with entering orders in the electronic medical record (EMR)?
   1. I have watched others place admission orders but do not feel comfortable doing so by myself.
   2. I would feel comfortable placing admission orders synchronously with my resident or attending (We place the orders together).
   3. I would feel comfortable placing admission orders by myself with constant supervision (The resident or attending watches me place all the orders).
   4. I would feel comfortable placing admission orders independently (to be pended and co-signed by the resident or attending later).
   5. If I had the EMR clearance to place orders independently, I would feel comfortable entering admission orders without someone double checking (no co-sign necessary).

**Rating Scale for Self-Assessment of Confidence in Order Entry Skills**

| Behavioral Anchor | Associated Point Value |
| --- | --- |
| I have watched others place admission orders but do not feel comfortable doing so by myself. | 1 |
| I would feel comfortable placing admission orders synchronously with my resident or attending (We place the orders together). | 2 |
| I would feel comfortable placing admission orders by myself with constant supervision (The resident or attending watches me place all the orders). | 3 |
| I would feel comfortable placing admission orders independently (to be pended and co-signed by the resident or attending later). | 4 |
| If I had the EMR clearance to place orders independently, I would feel comfortable entering admission orders without someone double checking (no co-sign necessary). | 5 |

Chen H, van den Broek WE, ten Cate O. The case for use of entrustable professional activities in undergraduate medical education. *Academic medicine*. 2015;90(4):431-436.
