# Supplementary material for: Enter and Discuss Orders and Prescriptions (EPA 4): A Curriculum for Fourth-Year Medical Students
Source: MedEdPORTAL. 2022 Jul 5;18:11263. doi: 10.15766/mep_2374-8265.11263 (PMC9253226; doi:10.15766/mep_2374-8265.11263)
Supplement: Supplementary file 1 — Facilitator Guide.docxCase 1.docxCase 2.docxCase 1 Rubric.xlsxCase 2 Rubric.xlsxOrder Entry Workshop Debrief.pptxSelf-Report Confidence Instrument.docxGraduate Self-Report EPA 4 Preparedness Item.docx [file mep_2374-8265.11263-s001.zip › A. Facilitator Guide.docx]

Order Entry Workshop Facilitator Guide

During this 75-minute workshop, designed to introduce important order entry skills, students will work in pairs to place admission orders for simulated patients in an educational Electronic Health Record (EHR). They will then engage in a large group debrief to consolidate their learning.

**Pre-work:**

This workshop presumes that all students already have a basic familiarity with the EHR at your institution, but minimal experience with placing admission orders. Prior to the session, students should receive some targeted instruction on the logistics of placing admission orders. We suggest either providing a narrated recording of the process of admission order entry for students to view asynchronously prior to the workshop, or spending the first 10 minutes of the workshop conducting a live demonstration of the key steps utilizing the educational EHR at your institution

**Materials:**

Computer / projector for facilitator

Personal computers for students, with appropriate software for using the educational EHR

List of Patient Names (clones) to facilitate each student working in a unique patient chart

Order Entry Rubrics for Cases 1 and 2.

If implementing this curriculum without access to an educational EHR, print paper copies:

- Cases 1 and 2
- Institutional order sets that may be relevant for cases 1 and 2
- Institutional clinical guidelines that may be relevant for cases 1 and 2
- Allow access to electronic decision support tools such as national clinical practice guidelines, pharmacy resources, or UptoDate©

**Timeline:**

10 minutes: Demonstrate the general process of placing admission orders using your institution’s educational EHR (if required).

40 minutes: Students divide into pairs to work on reviewing cases that have been loaded into the educational EHR. Students will each work with a partner who has the same patient case. However, each student will be assigned a unique patient clone to allow for simultaneous use of the patient records.

Each of the patient cases involves a patient who is currently in the emergency department and now requires admission to the student’s inpatient service. Students work collaboratively to place appropriate admission orders for their patients. Students should be encouraged to utilize any available resources they might typically access while caring for patients. This may include institution-specific evidence-based practice guidelines, or other resources. During this time facilitators circulate amongst the students to answer questions or facilitate learning and new insights amongst students.

10 minutes: Student pairs self-assess their final admission orders with an expert-developed rubric

15 minutes: Workshop Debrief

**The Cases:**

Case #1: A young patient with pyelonephritis in context of an obstructing kidney stone.

Case #2: A patient with inflammatory bowel disease, diarrhea, and syncope.

**The Rubrics:**

These rubrics are intended for learning (not assessment) during the workshop. The rubrics should not be given to the students until after they feel their admission orders are complete. Then, they can use the rubrics to assess their orders for important errors, differences, or omissions. The rubrics address the critical elements within a case and allow for variation in clinical decision making. For instance, in case 2, points are deducted if students fail to demonstrate high value care principles and repeated labs that had just been drawn in the ED but no points are deducted for “could do actions” such as ordering a fluid bolus or obtaining orthostatic vitals. Each rubric designates must order, should order, could order, should not order, and must not order elements.

Case 1

Critical elements and actions

- Correct documentation of penicillin allergy
- Medication reconciliation
- Correct order for code status (Full)
- Orders NPO for diet – potential upcoming procedure
- Orders isotonic maintenance fluids
- Orders non-penicillin containing antibiotic for treatment of complex urinary tract infection
  - Levofloxacin or ciprofloxacin (institutional guideline.  Should modify to reflect local practice)
- Orders deep vein thrombosis (DVT) prophylaxis – heparin 5000 units SC Q8H (institutional preference due to higher risk category due to concurrent hormonal contraceptive use. Should modify to reflect local practice)
- Orders platelet monitoring – monitor for side effects of heparin use (institutional policy. Should modify to reflect local practice)
- Consults Urology for consideration of lithotripsy

Must not order and errors of omission

- Inaccurate or absent documentation of penicillin allergy
- Incorrect or incomplete medication reconciliation
- Orders incorrect code status or absent code status
- No vitals monitoring
- No DVT prophylaxis
- Orders penicillin containing antibiotic
- No orders for IV fluids or orders hypotonic fluids
- Orders unnecessary monitoring such as cardiac monitor or continuous pulse oximeter
- Orders duplicate labs or imaging recently obtained in ED

Case 2

Critical elements and actions

- Correct documentation of no known allergies
- Medication reconciliation
- Correct order for code status (Full)
- Orders vital signs monitoring
- Orders Up with Assistance Only – patient was recently syncopal
- Orders isotonic maintenance fluids
- Orders DVT prophylaxis – heparin 5000 units SC Q8H (institutional preference, despite blood streaked stools given hypercoagulable state and high risk for clot with IBD history. Should modify to reflect local practice)
- Orders infectious stool studies to evaluate etiology of bloody diarrhea
- Orders Gastroenterology consult

Clinical variation

- Treatment of c. diff (with vancomycin or fidaxomicin) or IBD flair (steroids) – could reasonably be initiated empirically or deferred until c. diff testing results

Must not order and errors of omission

- Inaccurate or absent documentation of no known allergies
- Incorrect or incomplete medication reconciliation
- Should not order omeprazole due to increased risk of c. diff recurrence
- Orders incorrect code status or absent code status
- No vitals monitoring
- No DVT prophylaxis
- No orders for IV fluids or orders hypotonic fluids
- Orders unnecessary monitoring such as cardiac monitor or continuous pulse oximeter
- Orders duplicate labs or imaging recently obtained in ED

**The Debrief:**

The provided slide presentation may be used to help guide the discussion regarding the order entry exercise. As half of the students in the group will have completed case #1, and the other half will have completed case #2, consider encouraging students to share lessons learned from each of the cases. Facilitate discussion regarding order sets, clinical decision-making tools, high value care, and EHR alerts. Refer to the case rubrics to facilitate discussion on individual orders for the cases. Address the “why” and rationale, review the orders placed by the students, answer any questions about specific orders from each case and provide clinical reasoning for why some orders were correct and others not. Explain where multiple different choices may have been correct.
