# Supplementary material for: Enter and Discuss Orders and Prescriptions (EPA 4): A Curriculum for Fourth-Year Medical Students
Source: MedEdPORTAL. 2022 Jul 5;18:11263. doi: 10.15766/mep_2374-8265.11263 (PMC9253226; doi:10.15766/mep_2374-8265.11263)
Supplement: Supplementary file 1 — Facilitator Guide.docxCase 1.docxCase 2.docxCase 1 Rubric.xlsxCase 2 Rubric.xlsxOrder Entry Workshop Debrief.pptxSelf-Report Confidence Instrument.docxGraduate Self-Report EPA 4 Preparedness Item.docx [file mep_2374-8265.11263-s001.zip › H. Graduate Self-report EPA 4 Preparedness Item.docx]

**Graduate Self-report EPA4 Preparedness Item**

I feel prepared to enter and discuss orders and prescriptions.

1. Strongly Disagree
2. Disagree
3. Disagree/Agree Equally
4. Agree
5. Strongly agree
