# Supplementary material for: Enter and Discuss Orders and Prescriptions (EPA 4): A Curriculum for Fourth-Year Medical Students
Source: MedEdPORTAL. 2022 Jul 5;18:11263. doi: 10.15766/mep_2374-8265.11263 (PMC9253226; doi:10.15766/mep_2374-8265.11263)
Supplement: Supplementary file 1 — Facilitator Guide.docxCase 1.docxCase 2.docxCase 1 Rubric.xlsxCase 2 Rubric.xlsxOrder Entry Workshop Debrief.pptxSelf-Report Confidence Instrument.docxGraduate Self-Report EPA 4 Preparedness Item.docx [file mep_2374-8265.11263-s001.zip › Educational_Summary_Report_11263.pdf]

MedEdPORTAL® The AAMC Journal of Teaching and Learning Resources

Original Publication Open Access

Enter and Discuss Orders and Prescriptions (EPA 4): A Curriculum for Fourth-Year Medical Students

Nancy Liao, MD*, Cynthia Leung, MD, PhD, Jeff Barbee, DMA, MA, Gabrielle Gonzales, MD, Troy Schaffernocker, MD, Nick Kman, MD, Camilla Curren, MD, Kristen Lewis, MD

*Corresponding author: nancy.liao@nationwidechildrens.org

Abstract

Introduction: Order entry, entrustable professional activity (EPA) 4, is one of several EPAs that residency program directors identify as a weakness for PGY 1 residents. A multispecialty survey of program directors indicated that only 69% of interns could be trusted to enter and discuss orders and prescriptions without supervision. To address this gap, we developed a formative workshop for fourth-year medical students. **Methods:** Prior to the start of their subinternships, 366 fourth-year medical students engaged in an order entry workshop. Students performed chart reviews on electronic standardized patients within an educational electronic health record (EHR), placed admission orders, customized order sets, responded to safety alerts, utilized decision support tools, and incorporated high-value care considerations. Students used expert-validated rubrics to assess the quality of their admission orders and participated in a facilitated group discussion on key learning points. Finally, students participated in order entry, with all orders requiring cosignature by a supervising physician, during their clinical rotations. Students reported their confidence with order entry before and after the workshop and after the clinical rotation. **Results:** One hundred seventeen students completed the pre- and postworkshop surveys, and 99 went on to complete the postcourse evaluation. Students showed a statistically significant increase in their confidence level following the workshop. **Discussion:** Order entry is a critical, complex skill that requires deliberate instruction. This curriculum, which leverages the features of an educational EHR, can facilitate instruction, practice, and confidence gains regarding order entry prior to further application of these skills in the clinical environment.

Keywords
Order Entry, Electronic Health Record, Case-Based Learning, Chart Review, Clinical/Procedural Skills Training, Competency-Based Medical Education (Competencies, Milestones, EPAs), High-Value Care/Cost-Conscious Care

Educational Objectives

By the end of this session, learners will be able to:

1. Enter a complete set of admission orders, using an order set, for a patient being admitted from the emergency department to the hospital.
2. Recognize when to deviate from a standard order set when placing admission orders for a patient.
3. Respond to electronic health record safety alerts, including those for code status, deep venous thrombosis (DVT) prophylaxis, and medication allergies and interactions.
4. Utilize decision support tools such as institution-specific clinical practice guidelines, DVT risk assessment tools, and infection control recommendations to inform safe order writing.
5. Incorporate high-value care principles into order entry through the avoidance of unnecessary cardiac monitoring, redundant diagnostic testing, and standing daily lab orders.

Citation:
Liao N, Leung C, Barbee J, et al. Enter and discuss orders and prescriptions (EPA 4): a curriculum for fourth-year medical students. *MedEdPORTAL*. 2022;18:11263.
https://doi.org/10.15766/mep_2374-8265.11263

Introduction

In 2014, the AAMC published core entrustable professional activities (EPAs) for entering residency to help bridge the gap between undergraduate and graduate medical education.[1] The 13 core EPAs clearly define the skills and behaviors all medical school graduates should be expected to perform with indirect supervision on day one of residency.[1-3] These EPAs include the ability to enter and discuss orders and prescriptions (EPA 4). The

Copyright © 2022 Liao et al. This is an open-access publication distributed under the terms of the Creative Commons Attribution license.

2014 AAMC program director EPA survey revealed relatively low residency program director confidence in the ability of PGY 1 graduates of Liaison Committee for Medical Education–accredited U.S. medical schools to perform with only indirect supervision many of these activities, among them being EPA 4.[4] A multispecialty survey of program directors several years later similarly revealed program director perceptions that only 69% of interns were adequately prepared to perform the skills required for EPA 4.[5]

On cursory review, proficiency with EPA 4 may seem easily achievable. In fact, prior to the widespread adoption of electronic health records (EHRs) spurred by the Patient Protection and Affordable Care Act in 2014, the activities associated with entering orders and prescriptions were in some ways more straightforward.[6] However, with the incorporation of computer physician order entry (CPOE) and clinical decision support (CDS) tools into EHRs, true entrustment requires not only synthesis of clinical data, consideration of high-value care principles, and demonstration of advanced communication skills but also engagement with clinical pathways, algorithms, and decision support, as well as the efficient utilization of the EHR.[7-9] Additionally, while incorporation of CPOE into EHRs has demonstrated improvements in patient safety, including reductions in adverse drug events,[10,11] the results that EHRs achieve remain subject to the impact of human factors.[12]

Though development guides exist,[7,13,14] little has been published on the implementation of curricula targeting EPA 4. Existing EHR workshops for medical students focus on information gathering, documentation, and direct patient care skills.[15-17] Such workshops emphasize a cognitive approach to EHR use and teach students how to incorporate the EHR into the medical visit while navigating the doctor-patient relationship.[15-17] One prior study demonstrated increased student self-reported confidence in EHR skills such as data gathering, documentation, and handling unsolicited information with a plan including order entry.[17] Prior studies on CDS and EHR curricula for residents have demonstrated increased preparedness in ordering imaging appropriately and improved EHR proficiency, efficiency, and confidence.[9,15,18] Medical students report generally positive attitudes towards the EHR and are eager to utilize it.[9,19,20] They also report that placing orders during their clerkship years helps them learn what tests and treatments are needed by their patients, establish a sense of ownership for their patients, and receive direct feedback on their work.[21] Building upon prior studies that facilitated students' growth in EHR data gathering and single order entry, we designed a curriculum to teach the advanced skills inherent in EPA 4 within the context of placing complex admission orders during a required subinternship course. In doing so, we aspired to improve graduating medical students' abilities and confidence regarding these skills.

Methods

We designed and implemented a 75-minute order entry workshop for all fourth-year medical students at the beginning of their required inpatient subinternship rotation. This was one component of a half-day orientation to prepare new subinterns for clinical rotations. All participants had previously completed all core clinical rotations, during which they had learned general principles of high-value care and had frequently utilized the EHR for information gathering and documentation purposes. Participants had additionally utilized the EHR for entering individual orders (such as labs or medications) for cosignature but had rarely utilized the EHR for entering complex orders such as admission orders or order sets. All participants were also familiar with utilization of the educational EHR, which had been incorporated into other curricula earlier in their education.

Facilitators for the workshop included hospitalist faculty and residents with significant personal experience of EHR order entry. Facilitators were oriented to the workshop via a facilitator's guide (Appendix A). Prior to the workshop, all students asynchronously watched a brief screencast demonstrating the step-by-step process for reconciling medications and utilizing an order set to place admission orders. Medication reconciliation is considered a critical step for admitting a patient at our institution, and providers routinely engage in this process as part of order entry.

During the in-person component, students utilized their personal devices (laptops or tablets) to access the educational EHR, which mirrored the production environment, with active order sets, safety alerts, and direct links to decision support tools, but did not contain real patient information. To achieve high-fidelity simulation of clinical practice, students were allowed resident-level privileges permitting medication reconciliation, use of order sets, placement and discontinuation of orders, and dismissal of safety alerts. Epic (Epic Systems Corporation) and Cerner (Cerner Corporation), two of the most-used EHR systems in the country, both market academic EHR environments that could be used to facilitate replication of this curriculum. Most EHR systems used in academia are created and maintained by local technical teams since CPOE sets and other portions of the record specific to the institution must be hand entered.

Copyright © 2022 Liao et al. This is an open-access publication distributed under the terms of the Creative Commons Attribution license.

Preparation of the workshop required that our local educational EHR staff use the information in the patient cases (Appendices B and C) to create patient charts. After creating a master version of each patient case, staff created multiple clones of the simulated patient's chart so that each student could access their own individual patient without impacting the experience of others. Creating clones also allowed for easy reproduction of unedited versions of the charts throughout the year.

Pairs of students worked collaboratively on one of two patient cases, with each student placing admission orders for their simulated patient independently via mock CPOE. Case 1 (Appendix B) consisted of a patient presenting with acute nephrolithiasis complicated by pyelonephritis. Case 2 (Appendix C) consisted of a patient with a history of inflammatory bowel disease who presented to the emergency department with blood-streaked diarrhea, dehydration, and syncope. Both cases were entered into the educational EHR as if the patients were currently in the emergency department.

We designed the cases to facilitate engagement with critical features of EHRs. To successfully complete orders, students were required to gather and synthesize patients' information from the EHR. Next, the students identified and selected appropriate admission order sets and customized them to reflect the needs of their patients. Students were encouraged to use any available decision support tools, such as local and national clinical practice guidelines, pharmacy resources, or UpToDate (Wolters Kluwer). Case 1 included a patient with history of anaphylaxis to penicillin. Students were allowed to utilize resources to order an appropriate antibiotic for that patient. During the exercise, students also were required to demonstrate consideration of high-value principles. For example, case 2 included a patient with lab tests that had been drawn in the emergency department (type and cross, coagulation panel, complete blood count, chemistries, and glucose). These labs also appeared as preselected orders in the admission order set for inflammatory bowel disease. Students needed to modify the order set and unselect those orders to avoid duplicating labs that had been already obtained. While entering admission orders, students also completed medication reconciliation and responded to EHR alerts related to medication interactions, patient allergies, and duplicate orders.

Throughout the exercise, facilitators circulated the room to help students, answer questions, and promote critical thinking. Once student pairs felt the admission orders were complete, faculty provided them with printed rubrics to critically self-assess the quality of their orders in comparison with those recommended by experienced clinicians (Appendices D and E). Course directors had previously created rubrics that had then been validated by a committee of practicing experts representing emergency medicine, critical care, and hospital medicine. The rubrics addressed the critical elements within a case and allowed for variation in clinical decision-making. For instance, in case 2, points were deducted if students failed to demonstrate high-value care principles and repeated labs that had already been drawn in the emergency department, but no points were deducted for could-do actions such as ordering orthostatic vitals. Following completion of this exercise, facilitators led a guided discussion related to order entry, highlighting prior experiences, challenges, and lessons learned (Appendix F). We set the expectation that students should engage with order entry during their clinical rotations. All clinical orders required cosignature by supervising physicians.

This order entry workshop was first introduced as a pilot program with a small cohort of students during the 2017-2018 academic year, and enhancements were made based on feedback from students and faculty facilitators. The workshop was instituted as a permanent part of the curriculum beginning in the 2018-2019 academic year. Starting in October 2018, students were asked to rate their own confidence with order entry prior to the start of the workshop, after the workshop, and at the end of the clinical course, utilizing an instrument derived from the modified Chen entrustment scale (Appendix G).[22] This prospective entrustment supervision scale was chosen to help facilitate students' self-assessment of their future supervision needs based on their current confidence and skill.

As part of a routine curricular evaluation process started at our school in the 2015-2016 academic year, students responded to a yearly questionnaire regarding their preparedness to perform the 13 core EPAs upon completion of medical school. Students were asked their level of agreement with the statement "I feel prepared to enter and discuss orders and prescriptions" based on a 5-point Likert scale (ranging from *Strongly Disagree* to *Strongly Agree*; Appendix H).

Results

A total of 366 fourth-year medical students participated in the EPA 4 order entry workshop during 2018-2019 and 2019-2020. A total of 218 students had the opportunity to complete preworkshop, postworkshop, and postcourse surveys. Of those students, 117 consented to participate in the study and completed the pre- and posttest surveys. The average preworkshop score was 2.4 ($SD = 1.0$), and the average postworkshop score was 3.5 ($SD = 0.7$). All assumptions for

Copyright © 2022 Liao et al. This is an open-access publication distributed under the terms of the Creative Commons Attribution license.

a paired *t* test were checked and met. We found a significant difference between the pre- and postworkshop scores, $t(116) = 10.96$, $p < .001$, $d = 1.01$, 95% CI: 0.79-1.24. The effect size is considered large according to the guidelines set by Cohen.[23]

An additional analysis was completed on the 99 students who consented to the study and completed all three of the preworkshop, postworkshop, and postcourse surveys. The distribution of responses is shown in the Figure. The average preworkshop score was 2.4 ($SD = 1.1$), and the average postworkshop score was 3.5 ($SD = 0.7$). Students reported an average confidence score of 3.6 ($SD = 0.8$) on the postcourse evaluation. Mauchly's test indicated that the assumption of sphericity had been violated, $\chi^2(2) = 6.85$, $p < .05$. Therefore, we adjusted the degrees of freedom using the Huynh-Feldt correction ($\varepsilon = .95$). The results showed a significant difference between test scores depending on the iteration, $F(2, 186) = 72.53$, $p < .001$. According to Cohen's guidelines,[23] the effect size was large ($\eta^2 = .27$). Using a Bonferroni multiple-comparison procedure, we found significant differences between the preworkshop and postworkshop scores ($p < .01$), as well as between the preworkshop and postcourse results ($p < .01$). There was not a significant difference between the postworkshop and postcourse results ($p = .37$; Table).

In addition, we analyzed students' responses to the item "I feel prepared to enter and discuss orders and prescriptions" on the graduate questionnaire. Those who participated in this curriculum and consented to participate in research (for 2018-2019 and 2019-2020, $n = 248$) were compared with a historical

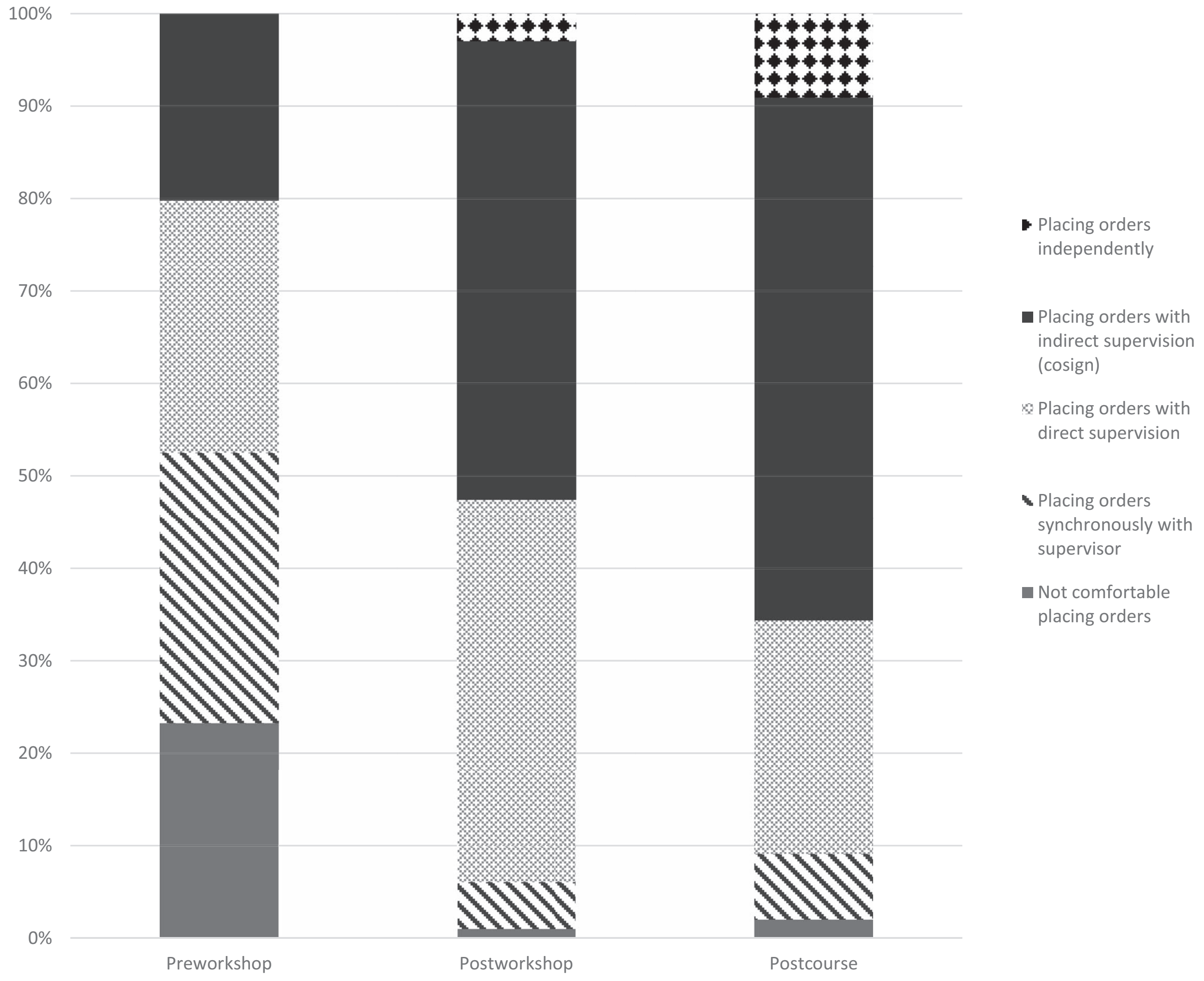

Figure. Students' self-reported comfort level with order entry at various time points ($n = 99$).

Copyright © 2022 Liao et al. This is an open-access publication distributed under the terms of the Creative Commons Attribution license.

Table. Results of the One-Way Repeated-Measures Analysis of Variance

Source	Sum of Squares	*df*	Mean Sum of Squares	*F*	*p*	η^2	95% η^2 CI
Test (within subjects)	83.4	2[a]	41.7	72.53	<.001	.27	0.17-0.36
Students (between subjects)	111.4	98	1.1				
Error	112.6	186[a]	0.6				
Total	307.4						

[a]Corrected degrees of freedom using the Huynh-Feldt adjustment.

control group of students who completed the course before the curriculum or pilot existed (for 2015-2016 and 2016-2017, $n = 277$). We implemented an independent samples *t* test after verifying all assumptions had been met. The mean score of students who participated in the order entry workshop was 4.2 ($SD = 0.8$), whereas students in the historical comparison had a mean score of 3.8 ($SD = 0.9$). Students who participated in this curriculum had significantly higher scores than the historical group of students, $t(517) = 4.82$, $p < .01$, $d = 0.42$.

Discussion

Preparation of students to perform EPA 4 skills is a core expectation of undergraduate medical education institutions yet has proven challenging, as evidenced by residency program director observations.[4,5] In the context of a health care system that increasingly relies upon EHRs for its CPOE and CDS functionalities, our curriculum provides undergraduate medical educators with the tools and framework to start preparing their students to effectively use these tools in order entry. In this formative workshop, students familiar with institutional EHR use and entering sporadic orders were tasked with entering a complete admission order set in a collaborative setting before their subinternship rotations. The workshop allowed students to gain confidence and experience prior to supervised practice. The educational EHR environment allowed for utilization and customization of order sets and engagement with CDS in real time. Explicit discussion of high-value care was also incorporated into the workshop.

The success of the curriculum is demonstrated by the statistically significant increase in students' levels of confidence with order entry. Students also reported that the curriculum motivated them to seek additional opportunities to enter admission orders during their subinternships, promoting more supervised practice and actual patient care experience. The self-reported increase in confidence did not necessarily equate to improved skill and quality of order entry. Because the workshop was intended to be formative, we did not collect the students' self-graded rubrics after the exercise to assess their accuracy. However, we hypothesize that translation of new knowledge and attitudes into skill will occur in the context of the clinical environment. Sarkar and colleagues found that deliberate practice with feedback in performing EHR skills resulted in improved accuracy of single orders placed by students.[24] Additionally, 87% of graduates from two of the participating Core EPA Pilot Project institutions were deemed to be prepared to perform EPA 4 skills by their residency program directors.[4] These studies suggest that intentional instruction and feedback can result in entrustment.

We propose that this workshop be delivered once, prior to the clinical experience, and serve as a primer for order entry. Requirements for cosignature of student orders by a supervising physician in the real world help to facilitate frequent direct observation, feedback, and possibly assessment of this skill. At institutions where students do not have order entry privileges in the clinical setting, we suggest multiple iterations of the workshop. To increase comfort and basic order entry skill, we recommend sessions at the beginning and end of a clinical rotation, with each student placing admission orders for both cases. For reinforcement and possibly to address knowledge attrition, the sessions should be repeated multiple times throughout the academic year, during capstone courses, and during residency preparation boot camps.

We also attempted to look at the additional impact of clinical experience on confidence with order entry. We compared the postworkshop and postcourse confidence levels and saw no significant difference, suggesting that the workshop alone was robust enough to build confidence and that real-life practice did not further increase this confidence.

Lessons learned include the necessity of rigorous case design and meticulous manual development of the cases in the educational EHR. Cases required not only complete patient data but also elements that would lead students to engage with CDS, alerts, and other EHR functionalities to drive accurate order choices and best patient management. Rubric development and validation by experienced clinicians with a variety of areas of expertise were necessary but time-consuming steps. Rubrics needed to assess not only for the presence of critical orders but also for the presence of critically inappropriate orders while additionally allowing flexibility when alternative orders might be reasonable. Using rubrics to guide students' self-assessment

Copyright © 2022 Liao et al. This is an open-access publication distributed under the terms of the Creative Commons Attribution license.

saved on human resources. One or two faculty were able to efficiently lead this session.

Optimal adoption of this curriculum requires access to an educational EHR, highlighting the most significant limitation to the curriculum's generalizability. Academic EHR environments are a major institutional expense and may not exist at all medical schools. While Educational Objectives 1, 2, 4, and 5 could be reasonably accomplished using a paper-based workshop, high-fidelity simulation of Educational Objective 3 (responding to an EHR alert) requires an interactive educational EHR. An additional limitation is the need to create multiple patient clones for multiple patient cases at different time points throughout the academic year, which required frequent contributions from the technology team supporting our institution's educational EHR. Access to such support might also limit generalizability.

We were unable to include all the students who participated in the curriculum in our analysis since only a smaller subset provided consent to be part of the study. Also, we initially planned to deploy only preworkshop and postcourse confidence surveys. To better understand the direct impact of our curriculum, in October 2018 we added a postworkshop confidence survey. We had some loss of data due to the change in study design and the inability to retrospectively obtain postworkshop surveys from students who had already completed the clinical rotation.

Finally, student access to the live EHR at our institution affords them a different level of privileges than those afforded to residents and attendings. While admission order sets and various CDS tools are readily accessible to students in the live EHR (with any orders requiring cosignature by a physician), students do not have privileges to engage in medication reconciliation. Although students were provided resident-level privileges to practice these skills within the educational EHR during the order entry workshop, they were unable to realistically practice medication reconciliation within the clinical environment. The educational value of any skills workshop that cannot be followed up with additional practice in the clinical setting is likely to be tempered. Hence, we advocate for EHR optimization, which would allow for the safe, supervised engagement of students in medication reconciliation during their clinical rotations.

This curriculum has also been adapted into a virtual workshop, utilizing videoconferencing and small-group breakout rooms, as necessitated by the COVID-19 pandemic. As a next step, we plan to include the deliberate assessment of student order entry skills, both within the educational EHR and within the clinical environment in the context of direct observation. We also plan to seek feedback from residency program directors regarding graduates of this curriculum to better understand the curriculum's impact.

Appendices

A. Facilitator Guide.docx
B. Case 1.docx
C. Case 2.docx
D. Case 1 Rubric.xlsx
E. Case 2 Rubric.xlsx
F. Order Entry Workshop Debrief.pptx
G. Self-Report Confidence Instrument.docx
H. Graduate Self-Report EPA 4 Preparedness Item.docx

All appendices are peer reviewed as integral parts of the Original Publication.

Nancy Liao, MD: Assistant Professor, Department of Pediatrics, The Ohio State University College of Medicine, and Section of Hospital Medicine, Nationwide Children's Hospital; ORCID: https://orcid.org/0000-0002-7188-7871

Cynthia Leung, MD, PhD: Assistant Professor, Department of Emergency Medicine, The Ohio State University College of Medicine

Jeff Barbee, DMA, MA: Research Specialist, Office of Curriculum and Scholarship, The Ohio State University College of Medicine

Gabrielle Gonzales, MD: Third-Year Resident, Department of Pediatrics, The Ohio State University College of Medicine, and Pediatric Residency, Nationwide Children's Hospital

Troy Schaffernocker, MD: Assistant Professor, Department of Pulmonary Critical Care and Sleep Medicine, The Ohio State University College of Medicine

Nick Kman, MD: Professor, Department of Emergency Medicine, The Ohio State University College of Medicine

Camilla Curren, MD: Associate Professor, Department of Internal Medicine, The Ohio State University College of Medicine

Kristen Lewis, MD: Assistant Professor, Department of Internal Medicine, The Ohio State University College of Medicine, and Section of Hospital Medicine, Nationwide Children's Hospital

Disclosures
None to report.

Funding/Support
None to report.

Copyright © 2022 Liao et al. This is an open-access publication distributed under the terms of the Creative Commons Attribution license.

Prior Presentations

Liao N, Lewis K, Kman N. EPA 4 (enter and discuss orders and prescriptions): laying foundations for residency success in medical school. Poster presented at: Transition to Residency; October 3-5, 2019; Chicago, IL.

Liao N, Curren C, Schaffernocker T, Kman K, Lewis K. Enter and discuss orders and prescriptions—curricular integration of an entrustable professional activity for fourth year medical students. Virtual poster presented at: Learn Serve Lead: the AAMC Annual Meeting; November 16-18, 2020.

Ethical Approval

Reported as not applicable.

References

1. *Core Entrustable Professional Activities for Entering Residency: Curriculum Developers' Guide*. Association of American Medical Colleges; 2014.
2. Lyss-Lerman P, Teherani A, Aagaard E, Loeser H, Cooke M, Harper GM. What training is needed in the fourth year of medical school? Views of residency program directors. *Acad Med*. 2009;84(7):823-829. https://doi.org/10.1097/ACM.0b013e3181a82426
3. Lypson ML, Frohna JG, Gruppen LD, Woolliscroft JO. Assessing residents' competencies at baseline: identifying the gaps. *Acad Med*. 2004;79(6):564-570. https://doi.org/10.1097/00001888-200406000-00013
4. Amiel JM, Andriole DA, Biskobing DM, et al; Association of American Medical Colleges Core EPAs for Entering Residency Pilot Team. Revisiting the core entrustable professional activities for entering residency. *Acad Med*. 2021;96(7S):S14-S21. https://doi.org/10.1097/ACM.0000000000004088
5. Pearlman RE, Pawelczak M, Yacht AC, Akbar S, Farina GA. Program director perceptions of proficiency in the core entrustable professional activities. *J Grad Med Educ*. 2017;9(5):588-592. https://doi.org/10.4300/JGME-D-16-00864.1
6. Hsiao CJ, Hing E. Use and characteristics of electronic health record systems among office-based physician practices: United States, 2001–2013. *NCHS Data Brief*. 2014;(143):1-8. https://www.cdc.gov/nchs/data/databriefs/db143.pdf
7. Obeso V, Brown D, Aiyer M, eds; Core EPAs for Entering Residency Pilot Program. *Toolkits for the 13 Core Entrustable Professional Activities for Entering Residency*. Association of American Medical Colleges; 2017.
8. Mejicano G, Ryan M, Vasilevskis EE. *Core Entrustable Professional Activities for Entering Residency—EPA 4 Schematic: Enter and Discuss Orders and Prescriptions*. Association of American Medical Colleges; 2017.
9. Willis MH, Frigini LA, Lin J, Wynne DM, Sepulveda KA. Clinical decision support at the point-of-order entry: an education simulation pilot with medical students. *Acad Radiol*. 2016;23(10):1309-1318. https://doi.org/10.1016/j.acra.2016.01.020
10. Prgomet M, Li L, Niazkhani Z, Georgiou A, Westbrook JI. Impact of commercial computerized provider order entry (CPOE) and clinical decision support systems (CDSSs) on medication errors, length of stay, and mortality in intensive care units: a systematic review and meta-analysis. *J Am Med Inform Assoc*. 2017;24(2):413-422. https://doi.org/10.1093/jamia/ocw145
11. Classen DC, Holmgren AJ, Co Z, et al. National trends in the safety performance of electronic health record systems from 2009 to 2018. *JAMA Netw Open*. 2020;3(5):e205547. https://doi.org/10.1001/jamanetworkopen.2020.5547
12. Sittig DF, Singh H. Electronic health records and national patient-safety goals. *N Engl J Med*. 2012;367(19):1854-1860. https://doi.org/10.1056/NEJMsb1205420
13. Peters H, Holzhausen Y, Boscardin C, ten Cate O, Chen HC. Twelve tips for the implementation of EPAs for assessment and entrustment decisions. *Med Teach*. 2017;39(8):802-807. https://doi.org/10.1080/0142159X.2017.1331031
14. Brown DR, Warren JB, Hyderi A, et al; AAMC Core Entrustable Professional Activities for Entering Residency Entrustment Concept Group. Finding a path to entrustment in undergraduate medical education: a progress report from the AAMC Core Entrustable Professional Activities for Entering Residency Entrustment Concept Group. *Acad Med*. 2017;92(6):774-779. https://doi.org/10.1097/ACM.0000000000001544
15. Zavodnick J, Kouvatsos T. Electronic health record skills workshop for medical students. *MedEdPORTAL*. 2019;15:10849. https://doi.org/10.15766/mep_2374-8265.10849
16. Biagioli FE, Elliot DL, Palmer RT, et al. The electronic health record objective structured clinical examination: assessing student competency in patient interactions while using the electronic health record. *Acad Med*. 2017;92(1):87-91. https://doi.org/10.1097/ACM.0000000000001276
17. Cristiano JA, Jackson JM, Shen E, Williams DM, Ellis LR. Integrating the electronic health record into patient encounters: an introductory standardized patient exercise for preclinical medical students. *MedEdPORTAL*. 2022;18:11209. https://doi.org/10.15766/mep_2374-8265.11209
18. Stroup K, Sanders B, Bernstein B, Scherzer L, Pachter LM. A new EHR training curriculum and assessment for pediatric residents. *Appl Clin Inform*. 2017;8(4):994-1002. https://doi.org/10.4338/ACI-2017-06-RA-0091
19. Knight AM, Kravet SJ, Harper GM, Leff B. The effect of computerized provider order entry on medical student clerkship experiences. *J Am Med Inform Assoc*. 2005;12(5):554-560. https://doi.org/10.1197/jamia.M1839
20. Rouf E, Chumley HS, Dobbie AE. Electronic health records in outpatient clinics: perspectives of third year medical students. *BMC Med Educ*. 2008;8:13. https://doi.org/10.1186/1472-6920-8-13

Copyright © 2022 Liao et al. This is an open-access publication distributed under the terms of the Creative Commons Attribution license.

21. Knight AM, Kravet SJ, Kiyatkin D, Leff B. The effect of computerized provider order entry on medical students' ability to write orders. *Teach Learn Med*. 2012;24(1):63-70. https://doi.org/10.1080/10401334.2012.641490

22. Chen HC, van den Broek WES, ten Cate O. The case for use of entrustable professional activities in undergraduate medical education. *Acad Med*. 2015;90(4):431-436. https://doi.org/10.1097/ACM.0000000000000586

23. Cohen J. *Statistical Power Analysis for the Behavioral Sciences*. 2nd ed. Lawrence Erlbaum; 1988.

24. Sarkar A, Aggarwal A, Grigoryan L, et al. Improving student confidence with electronic health record order entry. *PRiMER*. 2021;5:23. https://doi.org/10.22454/PRiMER.2021.619838

Received: November 5, 2021
Accepted: April 20, 2022
Published: July 5, 2022

Copyright © 2022 Liao et al. This is an open-access publication distributed under the terms of the Creative Commons Attribution license.
